# Ventilation improvement and evaluation of its effectiveness in a Japanese manufacturing factory

**DOI:** 10.1038/s41598-022-22764-2

**Published:** 2022-10-21

**Authors:** Hiroko Kitamura, Yo Ishigaki, Hideaki Ohashi, Shinji Yokogawa

**Affiliations:** 1 Occupational Health Training Center, University of Occupational and Environmental Health, Japan, 1-1 Iseigaoka, Yahatanishi-Ku, Kitakyushu, Fukuoka, 807-8555 Japan; 2 Graduate School of Informatics and Engineering, University of Electro-Communications, Chofu, Tokyo, Japan; 3 Info-Powered Energy System Research Center (I-PERC), University of Electro-Communications, Chofu, Tokyo, Japan

**Keywords:** Environmental sciences, Health occupations

## Abstract

A coronavirus disease 2019 (COVID-19) cluster emerged in a manufacturing factory in early August 2021. In November 2021, we conducted a ventilation survey using the tracer gas method. Firstly, we reproduce the situation at the time of cluster emergence and examined whether the ventilation in the office was in a condition that increased the risk of aerosol transmission. Secondly, we verified the effectiveness of the factory’s own countermeasure implemented immediately after the August cluster outbreak. Furthermore, we verified the effectiveness of several additional improvement measures on the factory’s own countermeasures already installed in August. Under the conditions of the cluster emergence, the air changes per hour (ACH) value was 0.73 ACH on average. The ACH value was less than 2 ACH recommended by the Ministry of Health, Labour, and Welfare, suggesting an increased risk of aerosol transmission. The factory’s own countermeasures taken immediately in August were found to be effective, as the ACH value increased to 3.41 ACH on average. Moreover, it was confirmed that additional improvement measures on the factory’s own countermeasures increased the ACH value to 8.33 ACH on average. In order to prevent the re-emergence of COVID-19 clusters due to aerosol infection in the office, it was found that while continuing the factory’s own countermeasure, additional improvement measures should also be added depending on the number of workers in the room. In a company, it is important that workers themselves continue to take infection control measures autonomously, and confirming the effectiveness of the measures will help maintain workers’ motivation. We believe it is helpful that external researchers in multiple fields and internal personnel in charge of the health and safety department and occupational health work together to confirm the effectiveness of conducted measures, such as in this case.

## Introduction

Since a case of unexplained pneumonia was reported in Wuhan City, China, in December 2019, coronavirus disease 2019 (COVID-19) has spread across the globe. By mid-December 2021, at least 272,819,000 global COVID-19 cases were reported, and 5,636,000 people were confirmed dead as a result. On January 8, 2020, the United States Center for Disease Control (CDC) officially announced that the cause of lung inflammation related to the seafood market in Wuhan, China, was the SARS-CoV-2 virus.

With any exhalation (quiet breathing, heavy breathing, talking, singing, exercising, coughing, sneezing, etc.), people emit various sizes of droplets. In patients with respiratory infections, droplets contain viruses, and droplets exhaled into the atmosphere are thought to carry the virus and transmit the infection. Xie et al.^1^ showed that 60–100 µm droplets would totally evaporate before falling 2 m away, 60–100 µm droplets would be carried more than 6 m when exhausted at a velocity of 50 m/s (sneezing), more than 2 m at a velocity of 10 m/s (coughing), and less than 1 m at a velocity of 1 m/s (breathing). Initially, infection with SARS-CoV-2 was considered to be caused by exposure to large droplets containing the virus. The transmission way was thought to be contact and droplet infection. Ong et al.^2^ investigated the contamination of the air, surface environment, and personal protective equipment by SARS-CoV-2 in rooms where symptomatic COVID-19 patients were admitted, and reported that swabs collected from the exhaust vents were real-time transcriptase-polymerase chain reaction (RT-PCR) targeting RNA-dependent RNA polymerase and E genes to detect the presence of SARS-CoV-2 positive. This suggested that small droplets containing the virus were suggested to be transferred by the airflows and deposited there. Hwang et al.^3^ reported an outbreak occurred between two vertical lines in an apartment, in which the virus was suspected to be spread through the air duct. This case showed the possibility of aerosol transmission. A cluster case in which the infection was suspected to be spread by airflow from an air conditioner in a poorly ventilated restaurant^4,5^, a cluster case in a relatively densely populated fitness club with an intense physical exercise^6^, a cluster case of transmission from an infected choir member to an attendee at a church without close physical contact^7^, a cluster case suspected an indirect transmission from asymptomatic patients in a shopping mall^8^. Considering these cluster cases, it was suspected that smaller droplets floated in the air for an extended period spread infection rather than being transmitted by contact or droplet transmission. On May 7, 2021, based on the scientific knowledge accumulated to date, the CDC declared that the transmission routes of SARS-CoV-2^9^ are as follows: (1) aerosol transmission (inhalation of air carrying very small fine droplets and aerosol particles that contain infectious viruses), (2) droplet transmission (deposition of virus carried in exhaled droplets and particles onto exposed mucous membranes (i.e. splashes and sprays, such as being cough on)), and (3) contact transmission (touching mucous membranes with hands soiled by exhaled respiratory fluids containing viruses or from touching inanimate surfaces contaminated virus). The three aforementioned infection ways are not mutually exclusive. At the end of October 2021, the Japanese Ministry of Health, Labour, and Welfare (MHLW) stated that transmission occurs when fine particles (aerosols) in the air containing SARS-CoV-2 are inhaled; as such, the Ministry recommends ensuring proper ventilation to combat aerosol transmission.

Japan has “public health centers” which are public institutions under the jurisdiction of the MHLW. There are 469 such centers nationwide based on the Community Health Act. These public health centers provide a comprehensive professional and technical base that supports the health of residents by providing consultation on intractable diseases and mental health, implementing infection countermeasures, and conducting monitoring and guidance on pharmaceutical affairs, food hygiene, and environmental hygiene. They also form a base for health crisis management, such as preventing the spread of diseases in the event of a health crisis and disseminating relevant information. Public health centers conduct “active epidemiological investigation” on patients with confirmed COVID-19 detected in Japan following the Act on the Prevention of Infectious Diseases and Medical Care for Patients with Infectious Diseases. In these investigations, the patient is asked about their activities within 14 days before the onset of symptoms to estimate the source and route of infection. They are also asked about their activities 2 days before the onset of symptoms to identify close contacts.

In the office of a manufacturing factory in Fukuoka Prefecture, five confirmed cases of COVID-19 successively emerged within 1 week in early August 2021, and the infections were confirmed to be a cluster (a group of infected people whose infections are linked). The local public health center stated that "the infected people were concentrated inside the office, and although there are working electric fans inside, there are no vents through which the virus can escape, resulting in the spread of infection." Immediately, the employer of this factory installed the factory’s own countermeasures. At first, all office ventilation equipment was inspected, and any failures detected were repaired as soon as possible. Other measures included the relocation of electric fans to the opposite side of the room considering the airflow and making it a rule to open the room door when working inside. However, as mentioned above, the public health center investigation was based on interviews, and the office's ventilation when the cluster emerged was not evaluated. The level of ventilation after the factory’s own countermeasures was not evaluated either.

The objective of this study is to: (1) reproduce the environmental variables at the time of cluster emergence and examine whether the ventilation in the office increased the risk of aerosol transmission; (2) verify the effectiveness of the factory’s own countermeasures immediately taken in August; and (3) verify the effectiveness of additional improvement measures on the factory’s own countermeasure.

## Materials and methods

### Workplace conditions where the cluster emerged

**Figure 1:**
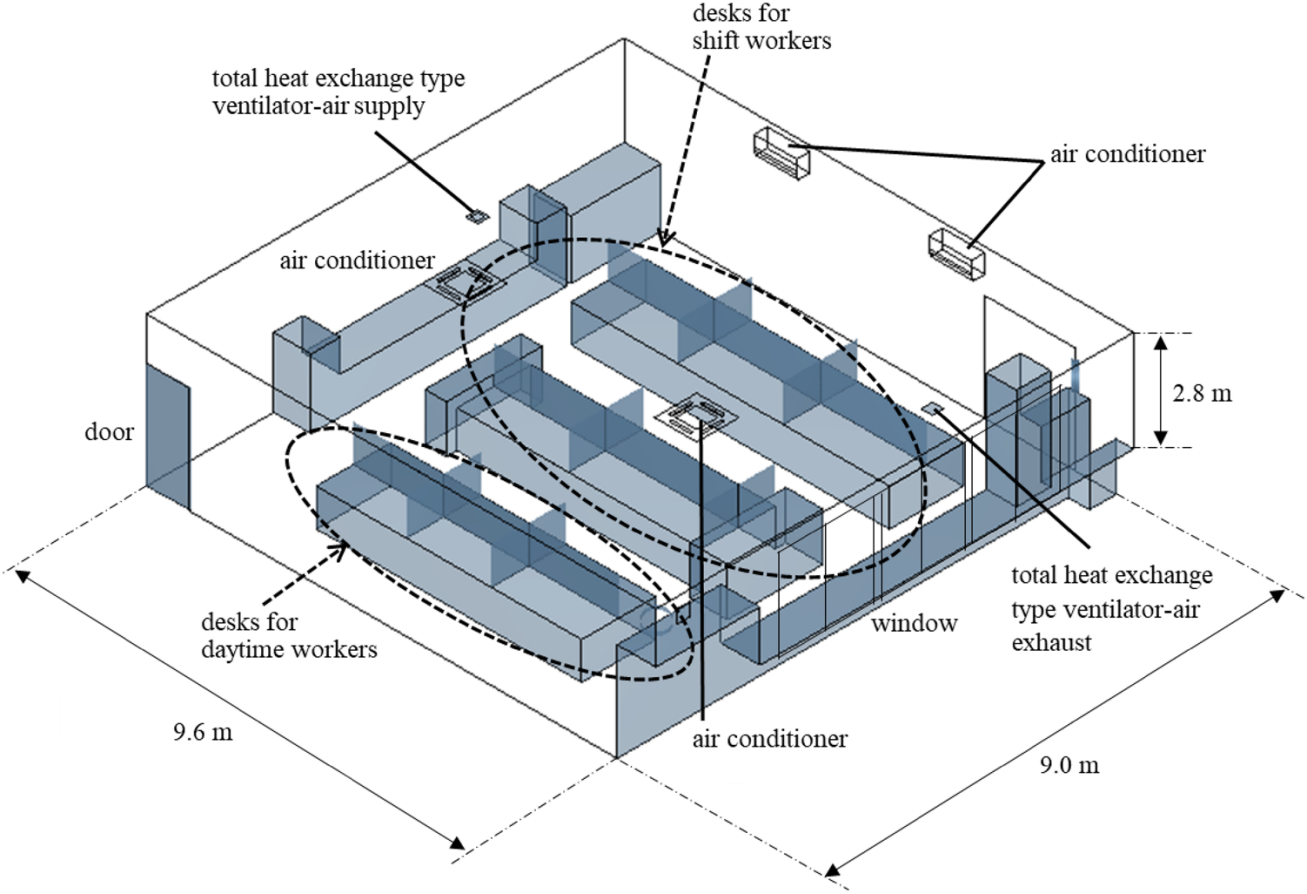
The layout of the workplace.

The affected workplace is a department managing information during production at the factory and performs desk work in the office and intervention on the operation site. It is managed by mixed groups of daytime workers and two groups with two shifts. On average, workers spend 70% of their time in the office and 30% in the field out of the 7 h and 45 min of work per day. At the time of the cluster’s emergence, 2–3 people were working in the daytime, and 16 people were working in shifts. As a measure against droplet transmission, 1.4 m high partitions had been installed between desks. The layout of the workplace was shown in Fig. 1.

### Status of cluster emergence

**Figure 2:**
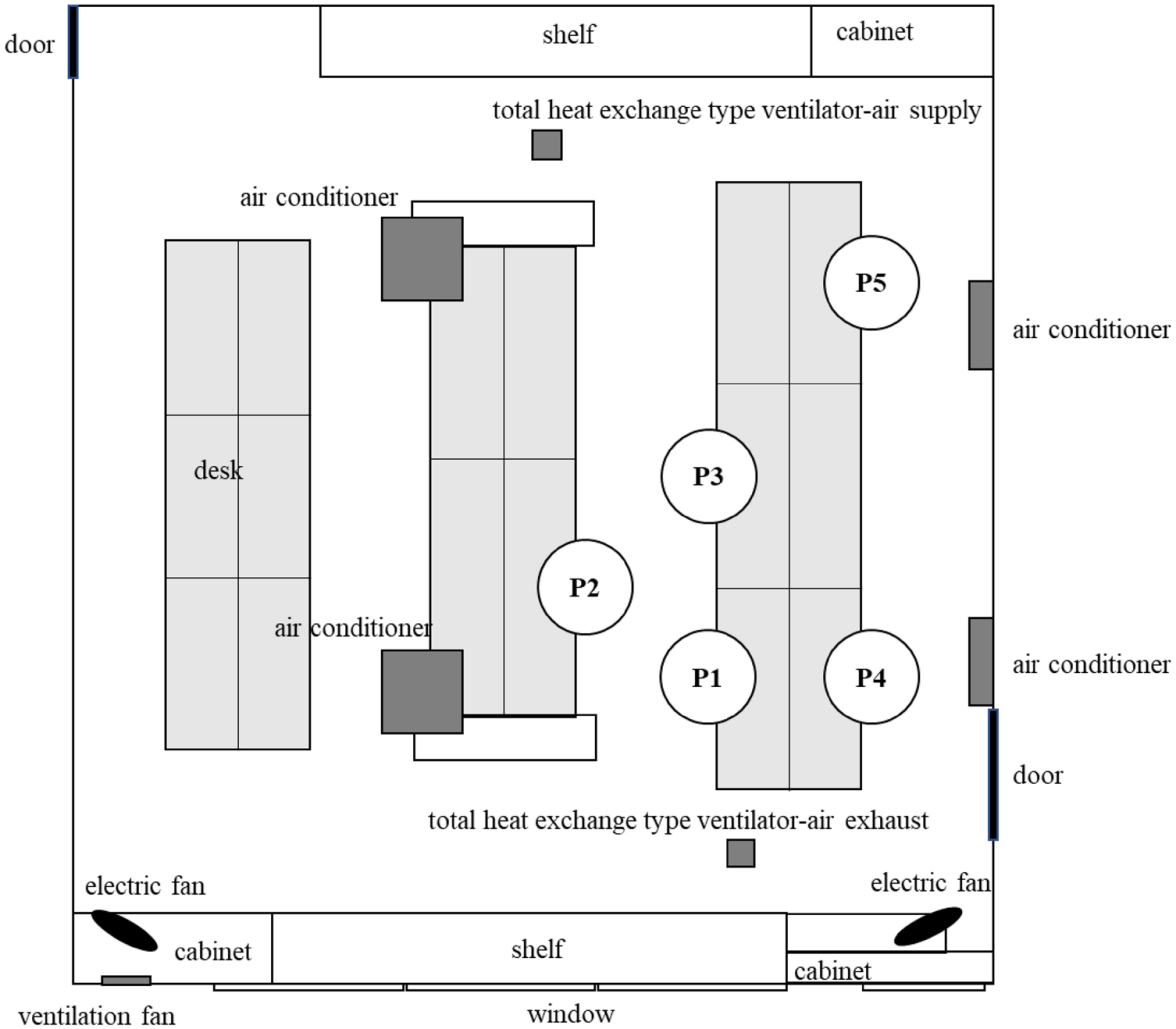
Desk placement of infected individuals.

**Figure 3:**
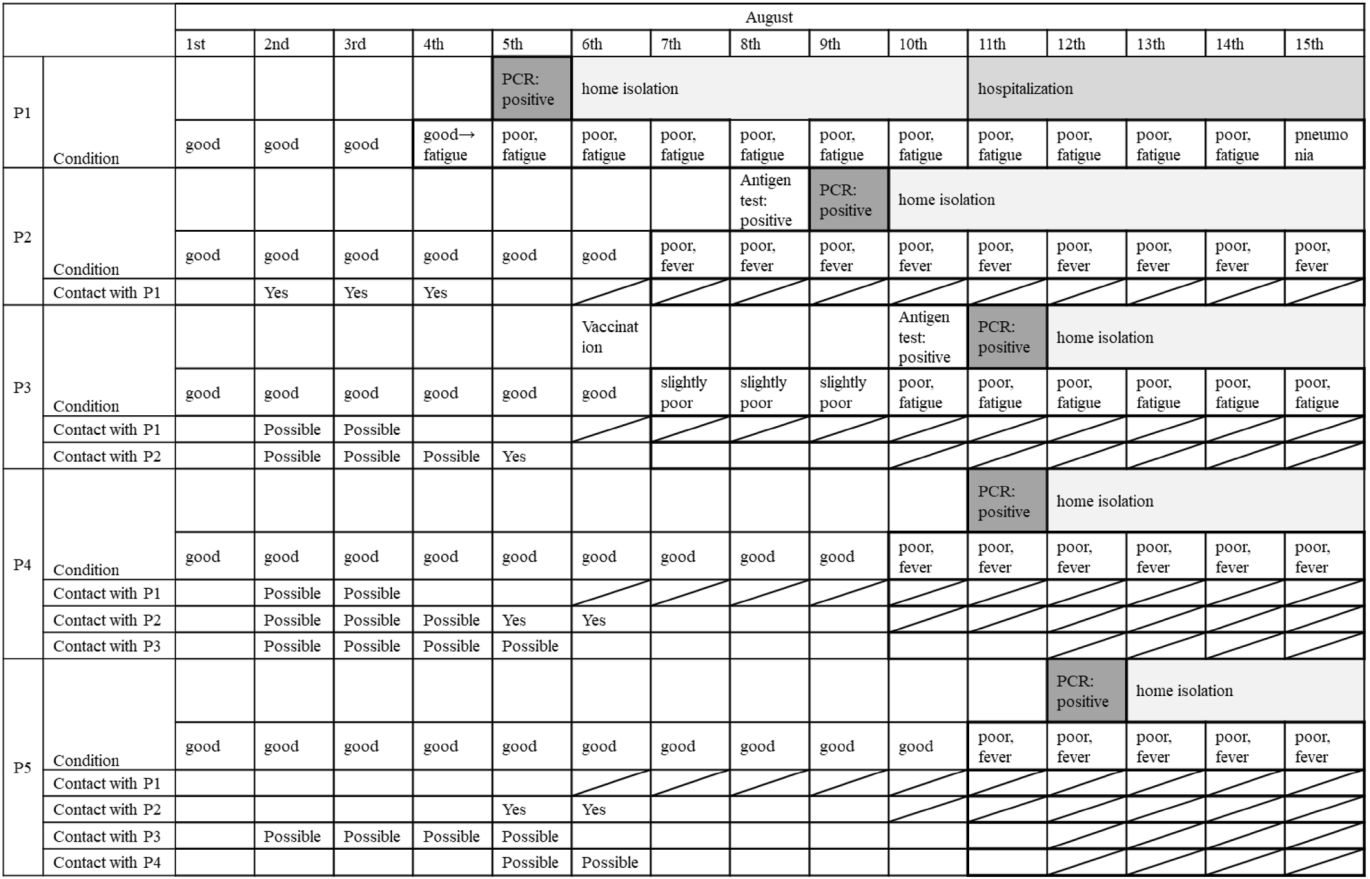
Time course of the emergence of infected patients.

**Figure 4:**
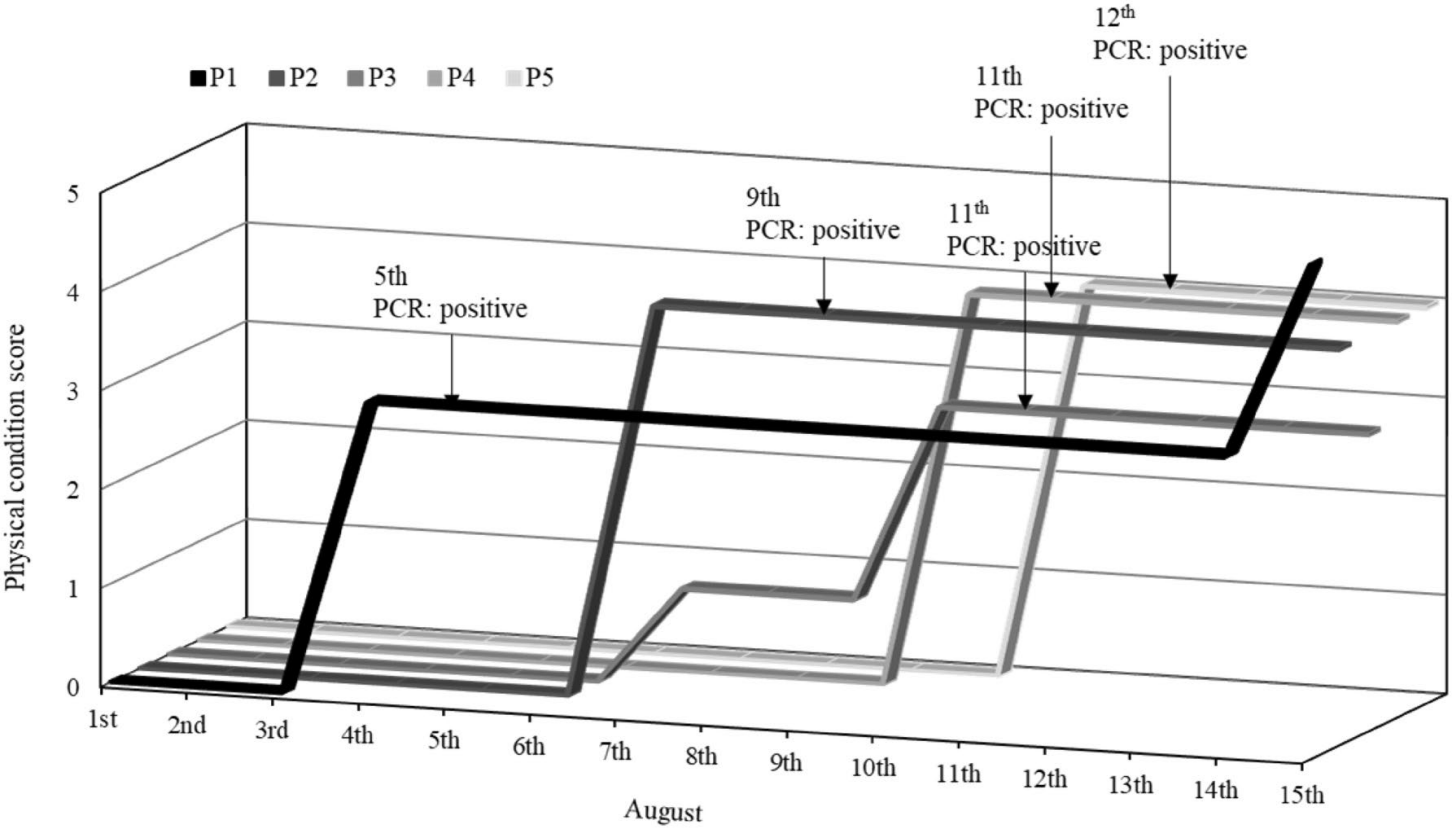
Change in physical condition score and time course for each patient.

On August 5, 2021, the first patient, indicated as P1 in Fig. 2, was confirmed by polymerase chain reaction (PCR), and then PCR-positive individuals emerged on August 7, 8, 11, and 12 within the same group (P2 to P5 in Fig. 2 in order). Patients were found only in one of the two groups of shift workers. Interviews with workers revealed the following: P1, P2, and P3 had many opportunities to talk to each other in close proximity in the aisles between desks, and P4 and P5 had many opportunities to be near other patients not only in the office but also outside of the office, and P1 to P5 often worked in close proximity and talked while they all looked at the same monitor. Figure 3 showed the time course of the emergence of infected patients, the physical condition of the patients, and the state of contact between patients. For the status of the contact, “Yes” was indicated when there was a clearly confirmed contact, and “Possible “ was indicated when there was a possibility of contact. Figure 4 showed the change in physical condition score and time course for each patient. Physical condition score was calculated as follows: good: 0, slightly poor: 1, poor:2, fatigue: 3, fever: 4, and pneumonia: 5. If more than one symptom was reported, the one with the higher number was adopted.

### Ventilation frequency survey method

#### Principle of the experiment

The ventilation frequency of buildings can be measured by the tracer gas method, which examines how marled air enters and exits the buildings. Tracer gas is generated in the room and its concentration changes over time are recorded. It is hypothesized that the air in the room and tracer gas diffuses instantly and uniformly. In this study, we used a step-down method, where the tracer gas generation would be stopped at a certain point and the ventilation rate was estimated from the time-lapse and concentration decay thereafter.

#### Carbon dioxide (CO_2_) sensor setting

**Figure 5:**
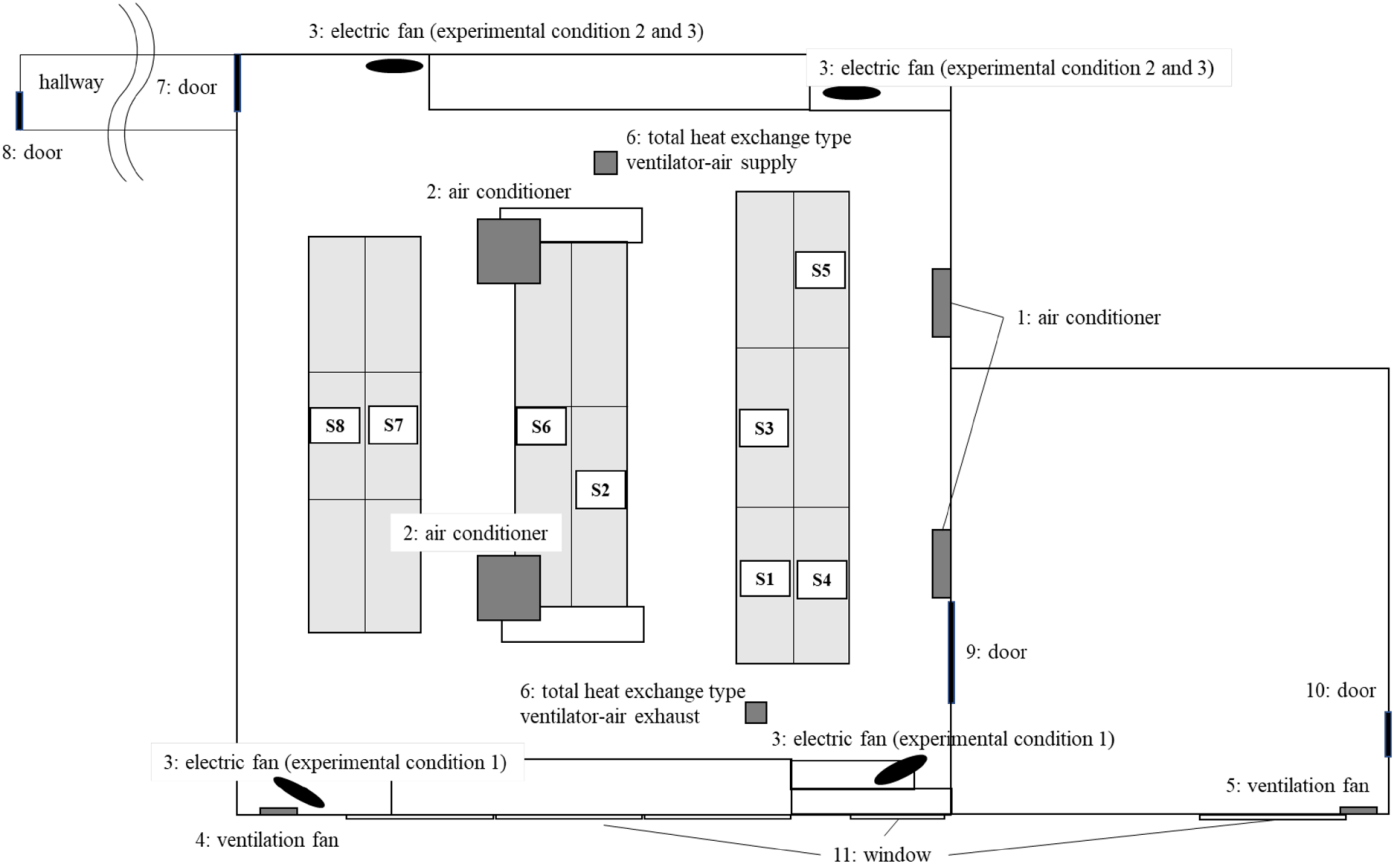
CO_2_ sensor location and experimental factors.

Eight CO_2_ sensors, indicated as S1 to S8 in Fig. 5, were installed. One sensor was installed for each section, separated by the infected person's desk and partition. The CO_2_ sensor measured and recorded the average CO_2_ concentration every 60 s. A non-dispersive infrared absorption-type CO_2_ sensor, TR-76Ui (T&D, Matsumoto, Japan), was used for the measurement. The TR-76Ui sensor can detect CO_2_ concentrations from 0 to 9,999 ppm, with an accuracy of ± 50 ppm (± 5%).

#### Measurement procedure

The office’s ventilation frequency (air changes per hour: ACH) was measured in three conditions; Experimental condition 1, Experimental condition 2, and Experimental condition 3, in that order.

- Experimental condition 1 (simulation of cluster outbreak).
  1. The office’s air conditioner and ventilation system were turned off, and all windows and doors were closed.
  2. Dry ice was then vaporized to fill the office with CO_2_, raising the CO_2_ concentration well above the background atmospheric CO_2_ concentration (400 ppm).
  3. Ventilation equipment, electric fans, windows, and doors were set under experimental condition 1 shown in Table 1.

**Table 1:** Experimental conditions.

Factor^a^		Experimental condition 1: simulation of cluster outbreak	Experimental condition 2: factory’s own countermeasures	Experimental condition 3: additional improvement measures on the factory’s own countermeasures
1	Air conditioner	On	On	On
2	Air conditioner	On	On	On
3	Electric fan^b^	On	On	On
4	Ventilation fan	Off	On	On
5	Ventilation fan	Off	Off	On
6	Total heat exchange type ventilator	Off	On	On
7	Door	30-mm open	Fully open	30-mm open
8	Door	Closed	Closed	30-mm open
9	Door	Closed	Fully open	Fully open
10	Door	Closed	Closed	30-mm open
11	Window	Closed	Closed	100-mm open

^a^Factors 1 to 11 in the Table 1 were linked to 1 to 11 in Fig. 4.

^b^Electric fans were placed in different locations in experimental condition 1 and experimental condition 2 and 3, as shown in Fig. 4.
  4. At the same time, all employees and researchers left the office because they were CO_2_ sources. The time at this point was considered as the ventilation start time.
  5. The CO_2_ concentration’s transition was monitored remotely, and the measurement was completed after confirming that the CO_2_ concentration had dropped sufficiently.
- Experimental condition 2 (factory’s own countermeasures).
  1. The office’s air conditioner and ventilation system were turned off, and all windows and doors were closed.
  2. Dry ice was then vaporized to fill the office with CO_2_, raising the CO_2_ concentration well above the background atmospheric CO_2_ concentration (400 ppm).
  3. Ventilation equipment, electric fans, windows, and doors were set under experimental condition 2 shown in Table 1.
  4. At the same time, all employees and researchers left the office because they were CO_2_ sources. The time at this point was considered as the ventilation start time.
  5. The CO_2_ concentration’s transition was monitored remotely, and the measurement was completed after confirming that the CO_2_ concentration had dropped sufficiently.
- Experimental condition 3 (additional improvement measures on the factory’s own countermeasures).
  1. The office’s air conditioner and ventilation system were turned off, and all windows and doors were closed.
  2. Dry ice was then vaporized to fill the office with CO_2_, raising the CO_2_ concentration well above the background atmospheric CO_2_ concentration (400 ppm).
  3. Ventilation equipment, electric fans, windows, and doors were set under experimental condition 3 in Table 1.
  4. At the same time, all employees and researchers left the office because they were CO_2_ sources. The time at this point was considered as the ventilation start time.
  5. The CO_2_ concentration’s transition was monitored remotely, and the measurement was completed after confirming that the CO_2_ concentration had dropped sufficiently.

### Estimation of ventilation frequency

Based on the data measured by the CO_2_ sensor, the ACH around each sensor was estimated based on the time-series change in CO_2_ concentration. Under the assumption that the volume of the incoming air and outgoing air were equal, that incoming air and the generated pollutants mixed immediately and completely, and that there was no trapping of pollutants on the walls, the number of indoor pollutants was described as (pollutants incoming with outdoor air) + (pollutants generated indoor) – (pollutants outgoing from the room) = (amount of change of pollutants in the room). The stable mass balance of well-mixed air could be described as:

1 $$V\frac{{dC_{t} }}{dt} = M + \lambda C_{0} - \lambda C_{t}$$

where $C_{t}$ is the concentration of indoor pollutants at time $t$, *M* is the number of pollutants generated in the room, $\lambda$ is the air ventilation rate [/h], and $C_{0}$ is the concentration in the absence of pollutant sources. The differential equation in Eq. (1) was solved as $C = C_{0}$ at $t = 0$ with the initial concentration of indoor pollutants as $C_{s}$, which is Seidel's formula^10^:

2 $$C_{i} = C_{0} + \left( {C_{s} - C_{0} } \right)e^{{ - \frac{Q}{V}\left( {i - s} \right)}} + \left( {1 - e^{{ - \frac{Q}{V}\left( {i - s} \right)}} } \right)\frac{M}{Q}$$

where $C_{i}$ is the concentration of indoor pollutants at time *i*, $C_{0}$ is the steady-state value in the absence of pollutants (400 ppm with atmospheric CO_2_ concentration as the background in this study), $C_{s}$ is the concentration of indoor pollutants at the ventilation start time, *V* is the room volume [m^3^], *Q* is the ventilation volume [m^3^/h], and *M* is the number of pollutants generated in the room.

When investigating the ventilation frequency, the room was unoccupied, and there was no other source of CO_2_. Therefore, if $M = 0$, Eq. (2) can be transformed as follows:

3 $$\ln \frac{{C_{t} - C_{0} }}{{C_{s} - C_{0} }} = - \frac{Q}{V}\left( {t - s} \right)$$

Based on the Eq. (3), the ventilation frequency $$\left( {{\raise0.7ex\hbox{$Q$} \!\mathord{\left/ {\vphantom {Q V}}\right.\kern-\nulldelimiterspace} \!\lower0.7ex\hbox{$V$}} \left[ {/h} \right]} \right)$$ was estimated from the slope of the natural logarithm of the CO_2_ concentration decrease ratio concerning the elapsed time from the ventilation initiation (ventilation time).

### Statistical analysis

We used the mixed-effect model to analyze the effects of experimental conditions on ventilation and the differences and trends in the ACH related to the CO_2_ sensor location. The dependent valuable was the ratio of the increase in CO_2_ concentration from the background to that at the ventilation initiation. We treated the ventilation time and interaction of ventilation time and experimental conditions and interaction of ventilation time and CO_2_ sensor location as fixed effects, and experimental condition and the sensor location as random effects. We used the statistical software JMP Pro Ver. 16 (JMP Statistical Discovery LLC.).

### Ethics declarations

This study was approved (approval number 21005) by the Ethics Committee on Experiments on Human Subjects of the University of Electro-Communications, Chofu, Tokyo, Japan. We confirmed that all experiments were performed in accordance with relevant guidelines and regulations. We measured CO_2_ concentrations as the “Three C’s” indicator, three C’s stand for “crowded places”, “close-contact settings”, and “confined and enclosed spaces”, in this study. We did not collect data linkable to specific individuals.

## Results

Table 2 shows the estimated ACH value calculated for each sensor under each experimental condition. The mean estimated ACH value of sensors 1–8 was 0.74 ACH when the cluster emerged: in experimental condition 1, 3.41 ACH after improvement measures in experimental condition 2, and 8.33 ACH for additional measures in experimental condition 3.

**Table 2:** Estimated air changes per hour (ACH) value per sensor.

Sensor	Estimated ACH value ± 95% CI^a^ (ACH)		
	Experimental condition 1	Experimental condition 2	Experimental condition 3
S1	0.726 ± 0.008	3.307 ± 0.033	8.673 ± 0.261
S2	0.728 ± 0.007	3.332 ± 0.040	8.479 ± 0.202
S3	0.721 ± 0.007	3.316 ± 0.034	8.332 ± 0.191
S4	0.726 ± 0.008	3.292 ± 0.031	8.509 ± 0.222
S5	0.754 ± 0.008	3.431 ± 0.080	8.598 ± 0.174
S6	0.761 ± 0.007	3.578 ± 0.043	8.458 ± 0.169
S7	0.726 ± 0.008	3.442 ± 0.059	7.666 ± 0.123
S8	0.759 ± 0.012	3.580 ± 0.062	7.932 ± 0.158
mean	0.738	3.410	8.331

^a^CI: confidence interval.

The effects of the ventilation time on the CO_2_ concentration and the interaction of the ventilation time and experimental conditions were statistically significant (*P* < 0.01). In other words, the CO_2_ concentration was significantly reduced by greater ventilation, and the ACH values were significantly different depending on experimental conditions (Table 3). On the other hand, the effect of the interaction of the ventilation time and the CO_2_ sensor location was not statistically significant (*P* = 0.35), and there was no obvious difference in the ACH values depending on the sensor location. Since the individual effects of experimental conditions and the CO_2_ sensor location, which are random effects, were not statistically significant (*P* > 0.05), respectively, it was unlikely that there was an individual effect other than the effects on the ventilation frequency (Table 3).

**Table 3:** Effects of experimental conditions and CO_2_ sensor location by mixed-effect model.

Factor	Number of parameters	DoF^a^ of numerator	DoF^a^ of denominator	F-value	*P*-value
**Fixed effects test**					
Ventilation time^b^	1	1	716.0	43,036.35	< 0.01
Interaction of Ventilation time and Experimental conditions	2	2	716.0	12,797.13	< 0.01
Interaction of Ventilation time and CO_2_ sensor location	7	7	716.0	1.13	0.35

Dispersion component	Estimated value	Standard error	95% CI^c^ Lower limit	95% CI^c^ Upper limit	Wald *P*-value
**Covariance parameter estimates for random effects**					
Experimental conditions	1.042	1.042	− 1.000	3.085	0.317
CO_2_ sensor location	0.000296	0.000177	− 0.000051	0.000642	0.0945
Residual error	0.00323	0.000171	0.00292	0.00359	
sum	1.046	1.042	0.284	40.398	

^a^DoF: Degrees of freedom.

^b^ventilation time means elapsed time from ventilation initiation.

^c^CI: confidence interval.

## Discussion

According to experimental condition 1, the ACH value at the time of cluster emergence was 0.73 ACH on average. Menzies et al.^11^ reported that a lack of ventilation is associated with an increased incidence of airborne infections; the higher the ventilation frequency, the higher the efficiency of air dilution, and the lower the risk of airborne infection. Menzies et al.^11^ also stated that a ventilation frequency of fewer than 2 times/h (equivalent to less than 2 AHC) is associated with the spread of tuberculosis, an airborne infection. With regard to tuberculosis, Toyota et al.^12^ reported that the ventilation frequency in junior high school classrooms in Japan where tuberculosis outbreaks occurred ranged from 1.6 to 1.8 times/h (equivalent to 1.6 ACH to 1.8 ACH). Wang et al.^13^ analyzed the tuberculosis cluster that occurred in an office in Taiwan and concluded that poor indoor ventilation resulted in a high CO_2_ concentration of over 1,300 ppm and led to the tuberculosis cluster. Du et al.^14^ also investigated the tuberculosis outbreak in underventilated university buildings in Taiwan and reported that exposing source tuberculosis cases in a CO_2_ concentration of over 1,000 ppm indoor environment was a significant risk factor for the spread of tuberculosis, and improving the ventilation rate to levels with CO_2_ concentration less than 1,000 ppm was associated with a 97% decrease in the incidence of new tuberculosis patients even though there were any contacts. In Guidelines for Preventing the Transmission of Mycobacterium tuberculosis in Health-Care Settings, 2005^15^, CDC showed the relationship between the ACH value and the airborne-contaminant removal efficiencies. When the ACH value was 2 ACH, 138 min were required to clear 99% of the air of airborne Mycobacterium tuberculosis, and 207 min were required for 99.9% of that. The risk of SARS-CoV-2 infection would be affected by the amount of virus exposed. The amount of virus exposed involves the presence of infected patients in the room who exhaled the virus. In some cases, a person entering or passing through the space after the infected person had left might be infected^5–8^. Van Doremalen et al.^16^ investigated the aerosol and surface stability of SARS-CoV-2 and reported that SARS-CoV-2 remained viable at least 3 h in aerosols with a reduction in infectious titer from 10^3.5^ to 10^2.7^ 50% Tissue Culture Infectious Dose (TCID_50_) per liter of air. Wang et al.^17^ described that the residence time of aerosols in still air can be estimated from Stokes’ law for spherical particles, for example, an aerosol of 100, 5, and 1 µm to fall for 1.5 m for 5 s, 33 min, and 12.2 h, respectively. Strictly speaking, there is a difference between airborne transmission and aerosol transmission; however, at the ACH value of only 0.73 ACH identified in experimental condition 1, the majority of aerosols containing the SARS-CoV-2 exhaled from infected patients were estimated to be floating for at least 3 h, which would increase the risk of aerosol transmission.

The mean ACH value in experimental condition 2 was 3.41 ACH, 4.7 times higher than when the cluster emerged. Under experimental condition 3, the mean ACH value was 8.33 ACH, 11.4 times higher than when the cluster occurred. The MHLW stated that to improve the ventilation of closed rooms with poor ventilation, the ventilation frequency should be increased to more than 2 times/h by opening windows without using mechanical ventilation (Ventilation to Improve Poorly Ventilated Enclosed Spaces in Commercial Facilities and others. https://www.mhlw.go.jp/content/10900000/000616069.pdf.). Under experimental condition 2, it was confirmed that the minimum ventilation frequency was secured. Moreover, the above-mentioned CDC guideline^15^ described that at 6 ACH, the time required for removal efficiency 99% was 46 min and 99.9% was 69 min, and at 12 ACH, 23 min for 99% and 35 min for 99.9%. For airborne infection isolation (AII) room, which was used to isolate patients infected with organisms spread via airborne droplet nuclei less than 5 µm in diameter, it was needed numerous ACH, more than 12 ACH for new construction as of 2011 and more than 6 ACH for construction before 2001^18^. It was found that in experimental condition 2, the ACH value was somewhat lower than the AII room constructed before 2011, and in experimental condition 3, the ACH value was higher than the AII room constructed before 2011 and lower than the newly constructed AII room.

The management standard of environmental sanitation for buildings for CO_2_ concentration according to the Act on Maintenance of Sanitation in Buildings is equal to or less than 1,000 ppm. According to Azuma^19^, based on the standard described in the WHO report “The physiological basis of health standard for dwellings^20^”, the management standard for CO_2_ concentration was set at 1,000 ppm in consideration of the health effects of CO_2_ when exceeding 1,000 ppm^21^. Since the volume of the office examined in this study was about 240 m^3^, in order to manage CO_2_ concentration in the office at 1000 ppm or less at the average ventilation frequency of 0.73 ACH and 3.41 ACH in experimental conditions 1 and experimental condition 2, respectively, it was estimated that the ventilation achieved under experimental condition 1 could cover five people while that of experimental condition 2 can cover 27 people. At the time of the cluster emergence, 18–19 people, including daytime workers and shift workers, were working simultaneously. The ventilation volume was less than 30% of the required amount, and this also indicated an increased risk of aerosol transmission. The ventilation volume achieved under experimental condition 2 would allow the original number of employees to work inside the office, and that under experimental condition 3 would allow 66 people to work in the office simultaneously. Of course, as stated in the JIS A1406^1974^ by Japan Industrial Standards Committee, the amount of CO_2_ that humans exhale depends on their level of physical activity^22^. As such, it would be safer to accommodate a smaller number of people than the above estimation if a heated discussion among a large number of people is to take place or if there is heavy activity to simulate the operation of a production line. If the door cannot be released to discuss sensitive content, it also would be safer to keep the number of people inside small and the meeting time as short as possible.

In our previous investigation of a cluster occurrence^23^, we found and reported that 1.6-m high vinyl sheet partitions installed between desks facing each other as a measure against droplet transmission blocked the office’s airflow, resulting in a section where air stagnated (vinyl sheet cluster). In the office space of this study, there were no differences in ventilation frequency depending on the sensor location, and there seemed no inhibitory effects of inappropriate partitions on ventilation. In the office, an electric fan created an airflow, as shown from the lower side to the upper right part of Fig. 1, which is considered to help scatter droplets containing the virus released from the first infected person without an outlet for air to escape to the outside. As a result, it was suggested that virus-containing droplets had gradually accumulated in the upper right part of the office shown in Fig. 1. An outbreak associated with air conditioning in a restaurant in Guangzhou, China^4^ was reported, and the “leeward cluster” was considered to have occurred because of very poor ventilation at this manufacturing factory as well. To prevent leeward clusters, when using an electric fan or blower to ensure adequate air circulation in a room, it is necessary to secure an air outlet and create outflow.

The following two proposals may be feasible for the operation of the office: First, when doing regular work, set the air conditioner, electric fan, ventilation fan, window, and door as experimental condition 2. If more people are required to work inside the office or during long discussions, add the countermeasure plan confirmed in experimental condition 3. Second, maintain experimental condition 2 and limit the number of people entering the room according to the work content.

Although this study confirmed the ventilation capacity of the office when there were no workers in the room, it did not ensure that ventilation would perform at its capacity when workers are active inside. The number of workers inside is not always constant, and the layout of the room may change in the future. Varying the number of workers inside and conducting ventilation experiments with tracer gas like this survey every so often is not realistic in terms of business continuity and infection control. A simple method to confirm that ventilation is ensured, that exhaled CO_2_ by workers is exhausted to the outside of the room, and that indoor air quality is controlled, is real-time monitoring using CO_2_ sensors. Indoor air quality control that visualizes CO_2_ concentration by real-time monitoring using CO_2_ sensors has attracted much attention^24,25^. There have been reports of real-time CO_2_ concentration monitoring being incorporated into ventilation control in school classeooms^26^, in a hospital^27^, in a gymnasium^28^, and in a long-distance sightseeing bus^29^. Visualization of CO_2_ concentrations can be used to validate that ventilation systems are working well, and can also be used to determine when to introduce additional measures to improve ventilation and to limit the number of people entering the room if the ACH value cannot be increased. We believe that CO_2_ concentration visualization can create an environment that is more flexible and allows workers to work with greater peace of mind.

It is essential to improve ventilation in the workplace, considering feasibility and sustainability and measures that can be put into practice without impeding work. The measures proposed in this study are based on workplace improvement activities and could be implemented continuously without difficulty. Of course, there are cases where improvements led by experts and researchers are necessary, but in the long run, the measures taken by workers who use the site daily are considered essential to prevent the recurrence of COVID-19 clusters.

## Limitation

In this study, we just investigated the ventilation effects in static environments. Even so, ventilation is an indirect assessment of aerosol transmission, as it observed the replacement of air in a room and assumes that aerosols were replaced together with the airflow. To investigate how the infection spread, it would be better to study the movement of the aerosol, if possible. A combination of factors, including particle size, room airflow, room temperature and relative humidity^30–32^ would influence on the actual behavior of infectious aerosols exhaled from a patient. The movement of individuals in the environment also would be taken into consideration^33–35^. We investigated the ventilation capacity of the office under the occurrence of cluster and in the two improved conditions and showed that the improvement measures maintained the sufficient ACH values indicated by the Japanese Act, the Act on Maintenance of Sanitation in Buildings, and CDC’s Guideline^15^, but further studies regarding aerosol behavior and the effects of workers inside are needed in the future. One idea is to consider the people working inside the room as the source of CO_2_ and to use a step-up method to estimate the ventilation frequency from the increase in the concentration of CO_2_ until it reaches a steady state.

## Conclusion

The survey of ventilation of the manufacturing factory where a COVID-19 disease cluster emerged revealed that: (1) the average ACH value in the office under the conditions of cluster emergence was 0.73 ACH, and the risk of aerosol transmission was considered to increase; (2) the factory’s own improvement countermeasures were found to be effective, as the average ACH value was increased to 3.41 ACH; and (3) the additional improvement measures on the factory’s own improvement countermeasures were much more effective because the average ACH value increased to 8.33 ACH, which was 11.4 times higher than that of the cluster emergence and 2.4 times higher than that of the factory’s own improvement. To prevent the spread of novel coronavirus infection in the workplace, employers would take measures considered to be effective. It is important that workers themselves continue to take infection control measures autonomously, and confirming the effectiveness of the measures will help maintain workers’ motivation. Additionally, it is helpful that external researchers in multiple fields and internal personnel in charge of the health and safety department and occupational health work together to confirm the effectiveness of conducted measures, such as in this case. Ventilation is just one measure against aerosol transmission. As measures against COVID-19 in the workplace, it is necessary to continue to avoid the three Cs (closed spaces, crowded places, and close-contact settings), ensure thorough hand hygiene, universal mask-wearing, and social distancing.

## Data Availability

The datasets generated during and/or analyzed during the current study are available from the corresponding author on reasonable request.
